# Changes in serum cholyglycine and prealbumin levels, glutamyl transpeptidase, and Alpha-fetoprotein after transcatheter arterial chemoembolisation combined with microwave ablation of liver cancer

**DOI:** 10.5937/jomb0-53123

**Published:** 2025-11-05

**Authors:** Kang Chen, Chaojie Zhang, Feihu Sun, Lei Sun, Chen Fan, Weidong Wang

**Affiliations:** 1 The Affiliated Wuxi Peoples Hospital of Nanjing Medical University, Department of Interventional Radiology, Wuxi Peoples Hospital, Wuxi Medical Center, Wuxi, 214000, People's Republic of China; 2 The Affiliated Wuxi Fifth Hospital of Jiangnan University, Department of Interventional Radiology, The Fifth Peoples Hospital of Wuxi, Wuxi, 214000, People's Republic of China

**Keywords:** PLC, TACE, MWA, liver function, SR, serum cholyglycine and prealbumin levels, glutamyl transpetidase, and alpha-fetoprotein, PLC, TACE, MWA, funkcija jetre, SR, nivoi holiglicina i prealbumina u serumu, glutamil transferaza, alfa-fetoprotein

## Abstract

**Background:**

This study aimed to evaluate the clinical outcomes of transcatheter arterial chemoembolisation (TACE) alone and in combination with microwave ablation (MWA) for patients with middle- or advanced-stage primary liver cancer (PLC) and analyse the causes of complications.

**Methods:**

A total of 100 patients with middle or advancedstage PLC were divided into two groups: the TACE group (TACEG, n = 50), which received TACE alone, and the combination group (CG, n = 50), which underwent TACE combined with MWA. Clinical parameters were evaluated before and after treatment, including the quality of life (QoL) assessed by the SF-36 score, serum liver function indices, treatment response, 1-year overall survival (OS) rate, and complication rates.

**Results:**

Compared to the TACEG, the CG demonstrated significantly higher SF-36 scores, objective response rate (ORR) (32% vs 50%), disease control rate (Dc R) (82% vs 90%), and 1-year OS (60% vs 84%), while exhibiting a lower 1-year complication rate (34% vs 16%). Additionally, post-treatment levels of cholyglycine (CG) and prealbumin (PAB) were significantly higher in the CG, whereas total bilirubin (TBil), direct bilirubin (DBil), alanine aminotransferase (ALT), aspartate aminotransferase (AST), alkaline phosphatase (ALP), gamma-glutamyl transferase (GGT), and alpha-fetoprotein (AFP) were significantly lower (all P&lt; 0.05).

**Conclusions:**

TACE combined with MWA is an effective and safe treatment for middle or advanced PLC, significantly improving liver function and postoperative survival rates.

## Abstract (sr)

**Uvod:**

Ova studija imala je za cilj da proceni kliničke ishode transkateterne arterijske hemoembolizacije (TACE) samostalno i u kombinaciji sa mikrotalasnom ablacijom (MWA) kod pacijenata sa primarnim rakom jetre (PLC) srednjeg ili uznapredovalog stadijuma, kao i da analizira uzroke komplikacija.

**Metode:**

Ukupno 100 pacijenata sa PLC-om srednjeg ili uznapredovalog stadijuma je podeljeno u dve grupe: TACE grupa (TACEG, n = 50), koja je primila samo TACE, i kombinovana grupa (CG, n = 50), koja je podvrgnuta TACE-u u kombinaciji sa MWA. Klinički parametri su procenjeni pre i nakon tretmana, uključuju i kvalitet života (QoL) procenjen SF-36 skorom, indekse funkcije jetre u serumu, odgovor na lečenje, stopu ukupnog preživljavanja (OS) tokom jedne godine i stope komplikacija.

**Rezultati:**

U poređenju sa TACEG, CG je pokazala značajno više SF-36 skorove, stopu objektivnog odgovora (ORR) (32% naspram 50%), stopu kontrole bolesti (DCR) (82% naspram 90%) i jednogodišnju stopu OS (60% naspram 84%), dok je stopa komplikacija tokom jedne godine bila niža (34% naspram 16%). Pored toga, nivoi holiglicina (CG) i prealbumina (PAB) nakon tretmana bili su značajno viši u CG, dok su ukupni bilirubin (TBil), direktni bilirubin (DBil), alanin aminotransferaza (ALT), aspartat aminotransferaza (AST), alkalna fosfataza (ALP), gama-glutamil transferaza (GGT) i alfa-fetoprotein (AFP) bili značajno niži (svi P&lt; 0,05).

**Zaključak:**

TACE u kombinaciji sa MWA predstavlja efikasan i bezbedan tretman za pacijente sa PLC-om srednjeg ili uznapredovalog stadijuma, značajno poboljšavajući i funkciju jetre i postoperativnu stopu preživljavanja.

## Introduction

PLC is a widespread malignant tumour with high incidence, high metastasis rate and high mortality [1]. According to the histological characteristics, PLC can be divided into cholangiocarcinoma, liver cancer (LC), and mixed carcinoma [2]. Due to the potential pathogenic characteristics of PLC, some patients have already developed to the middle or advanced stage when diagnosed, so they have lost the opportunity for surgical treatment. Therefore, TACE and other comprehensive interventional treatment modes have become the preferred treatment for some patients with PLC who cannot receive surgical treatment [3]. TACE is performed by identifying the feeding artery of the target lesion and injecting pre-mixed embolic agents or chemotherapeutic drugs into the artery, which then kills cancer cells, blocks the blood flow of the feeding artery, and promotes the necrosis and extinction of the target lesion after ischemia and hypoxia [4]. TACE has the advantages of minor trauma, a wide range of applications, and excellent therapeutic effects. As against interventional therapy alone, TACE can markedly prolong the survival time and improve the prognosis of patients with PLC. However, using TACE alone can cause various complications, and the long-term SR is not ideal [5] [6]. Moreover, TACE alone makes it easy to form collateral circulation and change the level of hemodynamics, so it also needs to be combined with MWA and other treatments.

MWA is also a commonly used minimally invasive treatment in clinical practice, and its mechanism of action is that local cell or tissue necrosis is caused by heat generated by microwave electromagnetic fields [7]. Under the action of microwave electromagnetic fields, the water molecules and protein molecules inside the target lesions produce high-speed vibration. The molecules collide and friction with each other, generating a high temperature of 60-150 in a short time. This causes denaturation and coagulation of proteins inside the cancer cells and necrosis, damaging the lesions [8]. MWA has the advantages of intense penetration, wide therapeutic range, apparent therapeutic effect, and real-time monitoring. Liu et al. [9] adopted magnetic resonance image-guided MWA in multifocal LC, with a local control rate of 98.7% after at least 1 year of follow-up. However, the clinical effect of TACE plus MWA in middle or advanced PLC is still controversial. Moreover, the complications of skin and sub-skin tissue injury caused by TACE are relatively rare.

Therefore, this article included patients with middle or advanced PLC, compared the outcome, liver function, QoL, and complications after TACE alone and TACE plus MWA, and analysed the causes and treatment of skin injury complications. The causes and treatment of skin injury complications were analysed. The patients were followed up for 1 year to analyse the OS. It provides a reference for further exploring the therapeutic effect of TACE plus MWA and improving the QoL and prognosis of patients.

## Materials and methods

### General information

A total of 100 patients with middle or advanced-stage primary liver cancer (PLC) were enrolled at Wuxi People's Hospital, Wuxi Medical Center, Nanjing Medical University, Wuxi 2021 and March 2024. Patients were divided into the TACE group (TACEG) and the combination group (CG), with 50 cases in each.

Inclusion criteria:

1. Patients met the diagnostic criteria outlined in the Clinical Diagnosis and Staging Criteria for PLC.
2. Diagnosis was confirmed by biopsy, with indications for MWA treatment.
3. Tumor stage classified as Ilb-IIIb according to TNM clinical staging criteria.
4. Expected survival time of more than six months.
5. Karnofsky Performance Score (KPS) 60.
6. Liver function classified as Child-Pugh grade A or B.

Exclusion criteria:

1. Presence of significant dysfunction in other vital organs.
2. Contraindications for surgery.
3. Allergy to medications used in the study.
4. Prior anti-cancer treatment before enrollment.
5. Intractable hepatic ascites or hepatic encephalopathy.
6. Inability to communicate effectively or poor treatment compliance.

This trial was approved by Wuxi People's Hospital, Wuxi Medical Center, Nanjing Medical University, and the Wuxi Ethics Committee, and all participants provided signed informed consent.

### Treatment methods

The TACEG adopted TACE alone after routine disinfection; according to the actual situation, the tumor-feeding artery was determined by angiography. After the Seldinger puncture, the tumour-feeding artery was perfused with the mixed emulsifier of 750-1,000 mg 5-fluorouracil (H12020675; Tianjin Taihe Pharmaceutical Co. LTD.; Specification: 20 mg), 40-60 mg cisplatin (H53020409; Kunming Guiyan Pharmaceutical Co., LTD.; 20 mg), 6-8 mg mitomycin C (H19999025; Haizheng Pfizer Pharmaceutical Co., LTD.; 10 mg) or 20 to 40 mg epirubicin (H20000496; Wuxi Pfizer Pharmaceutical Co., LTD.; 10 mg), 10 mL iodised oil (H37022398; Yantai Luyin Pharmaceutical Co., LTD.; 10 mL) for embolisation, twice a week, and once more after a 3-week interval. According to the condition of the subjects, an appropriate amount of gelatin sponge granule embolic agent was given (2014 No. 3771056; Shandong Weier Medical Technology Co., LTD.; 150-1,000 pm) to enhance embolisation therapy, until the mixed emulsifier was completely deposited in the lesion or the feeding artery of the tumour disappeared. It should pay close attention to the subjects' vital signs during the treatment process and inform the doctor in time if there is any abnormal situation. The treatment period was 1 month.

The CG adopted TACE plus MWA. MWA was performed 7 days after TACE. Colour Doppler ultrasound was used to determine the location, shape, size, and blood supply of the lesions before treatment, and 25 mg promethazine hydrochloride (H11021437; China Resources Double Crane Pharmaceutical Co., LTD.; 25 mg). Subjects were placed supine, routinely sterilised and laid with an auxiliary towel. CT scan was performed to detect the position and path of the puncture, the MWA needle was slowly inserted, and the CT scan was repeated to insert the needle into the target position. The ablation parameters were set according to the subjects' actual conditions. The preset power was 50-70 W, the time was 6-12 min, and the ablation range was 5-10 mm on the outer edge of the tumour. The vital signs of the subjects were closely monitored during the treatment, and the subjects were kept in bed for 24 hours after the operation. Routine treatments such as hemostasis, pain relief, infection prevention, and liver protection were performed in time. If the subjects had abnormal conditions, they should inform the doctor in time. The treatment period was 1 month.

### Observation indicators

Before treatment and 3 months following remedy, 5 mL of fasting peripheral venous blood was collected from the subjects in the morning. An automatic biochemical analyser detected TBil, DBil, ALT, AST, ALP GGT, CG, PAB, and AFP

Three months following the remedy, subjects' QoL was assessed using SF-36 developed by the Medical OutcomesStudy. SF-36 included 36 items, including physical function (PF), physical role (PR), bodily pain (Pp), total health (TH), vitality status (VS), social function (SF), emotional role (ER), and mental health (MH). Subjects with higher scores had better QoL.

Three months after the treatment, the therapeutic effect of the subjects was assessed, and enhanced CT or MRI examined the subjects. The complete response (CR) was defined as complete disappearance of the target lesions and no new lesions appeared; partial response (PR): the maximum diameter of the target lesion reduced by at least 30% than the baseline, maintaining for at least 1 month; stable disease (SD): reduction of the maximum diameter of the target lesion by less than 30% or an increase of less than 25% than the baseline; progressive disease (PD): increase of at least 25% in the maximum diameter of the target lesion or the appearance of new lesions. ORR and DCR were calculated using the following equation:

ORR=CR+PR (1)

DCR=CR+PR+SD (2)

The subjects were followed up for 1 year by telephone, WeChat, or outpatient visit. The OS of subjects was evaluated in months, from the beginning of treatment to the occurrence of death. In addition, the recurrence rate, the incidence of skin injury, and other complications were recorded.

### Data analysis

SPSS 23.0 statistical software was employed. Dichotomous data were expressed as n or %, and the chi-square test was adopted. Continuous variables were presented as mean±sd and analysed by student's t-test. Kaplan-Meier curve was drawn to describe the survival of subjects at 1 year after surgery, and the log-rank test was adopted. P <0.05 was considered statistically meaningful.

## Results

### Analysis of subjects' general data

Age, gender, course of disease, number of lesions, location of lesions, maximum diameter of lesions, positive rate of AFP, TNM stage, and Child-Pugh grade had no visible distinction in the subjects (P >0.05) (Table 1).

**Table 1:** Contrast of general data.

Information	TACE	TACE+MWA	*P*
Sample size	50	50	
Age	56.7±9.1	57.0±9.6	0.515
Gender, *n* (%)			0.430
Male	32 (64.0)	35 (70.0)	
Female	18 (36.0)	15 (30.0)	
Duration of disease, months	6.8±1.5	7.0±1.1	0.663
Number of lesions,* n* (%)			0.392
<3	33 (66.0)	31 (62.0)	
≥3	17 (34.0)	19 (38.0)	
Lesion location, *n* (%)			0.197
Left liver	28 (56.0)	24 (48.0)	
Right liver	22 (44.0)	26 (52.0)	
The maximum diameter of the lesion (cm)	3.2±0.8	3.1±0.6	0.654
AFP *n* (%)			
Positive	41 (82.0)	40 (80.0)	
Negative	9 (18.0)	10 (20.0)	
TNM stage, *n* (%)			0.706
Stage IIb	16 (32.0)	15 (30.0)	
Stage IIIa	23 (46.0)	25 (50.0)	
Stage IIIb	11 (22.0)	10 (20.0)	
Child-Pugh grade, *n* (%)			0.512
Grade A	28 (56.0)	26 (52.0)	
Grade B	22 (44.0)	24 (48.0)	

### Analysis of liver function of subjects

**Figure 1:**
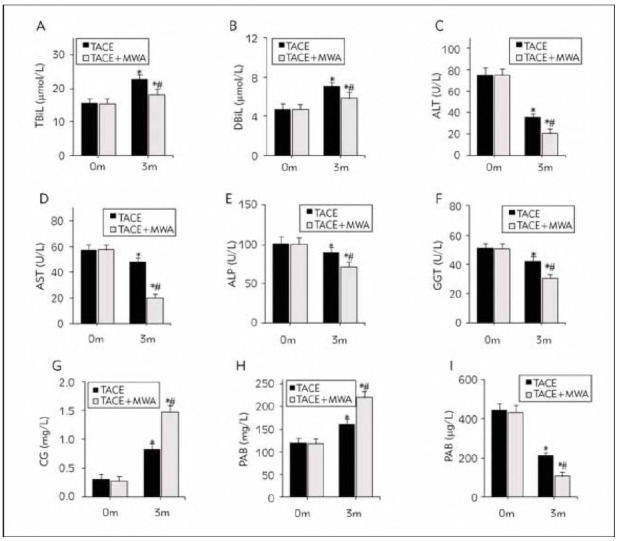
Comparison of serum levels of IL-1β, IL-6, TNF-α and CRP before and after intervention among the three group. (A-I display the serum levels of total bilirubin (TBil), direct bilirubin (DBil), alanine aminotransferase (ALT), aspartate aminotransferase (AST), alkaline phosphatase (ALP), gamma-glutamyl transferase (GGT), and alpha-fetoprotein (AFP) before and after the intervention. *Indicates a significant difference compared to the same group before the intervention; #indicates a significant difference compared to the TACEG group, with all P-values<0.05.)

Following the remedy, the serum TBil, DBil, CG, and PAB were markedly higher, and the serum ALT, AST, ALP GGT, and AFP were considerably lower in the TACEG and the CG as compared to before treatment. Following the remedy, the serum CG and PAB were markedly higher, while the serum TBil, DBil, ALT, AST, ALP GGT, and AFP were considerably lower in the CG as against the TACEG (all P <0.05) (Figure 1).

### QoL analysis of subjects

**Figure 2:**
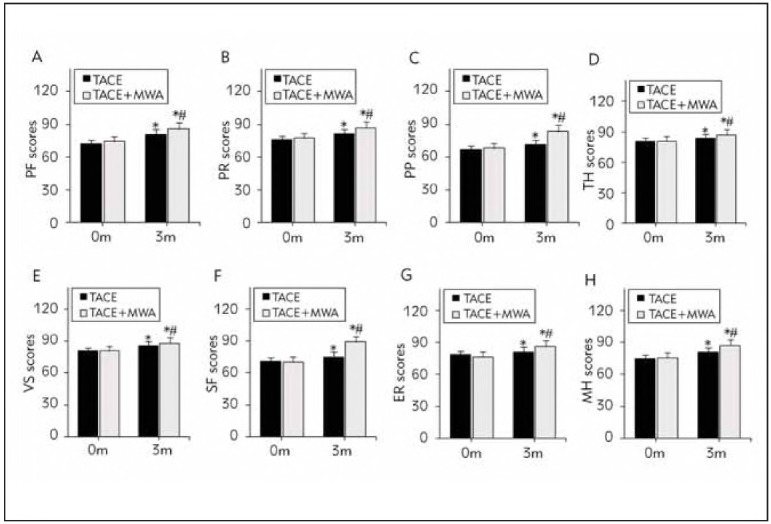
Comparison of SF-36 scores. (A-H illustrate the scores for physical function (PF), physical role (PR), physical pain (PP), total health (TH), vitality status (VS), social function (SF), emotional role (ER), and mental health (MH) before and after the intervention. *Indicates a significant difference compared to the same group before the intervention and a significant difference compared to the TACEG group, with all P-values <0.05.)

The scores of PF, PR, PP TH, VS, SF, ER, and MH in the TACEG and CG following remedy were markedly higher than before treatment. The scores were considerably higher in the CG as against the TACEG (all P <0.05) (Figure 2).

### Treatment effect analysis of subjects

**Figure 3:**
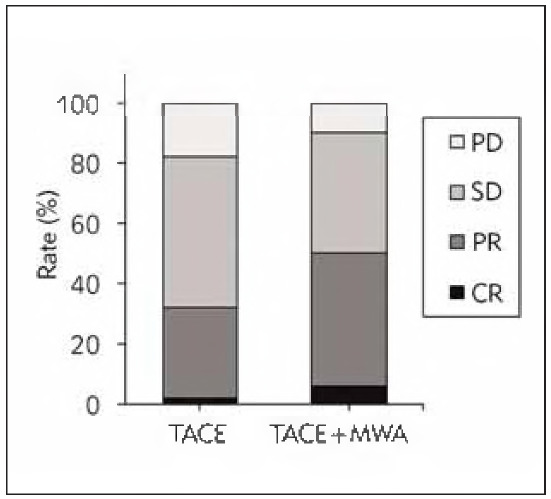
Contrast of treatment effects.

In the TACEG, 1 case (2.0%) achieved CR, 15 cases (30.0%) achieved PR, 25 cases (50.0%) achieved SD, 9 cases (18.0%) achieved PD, the ORR was 32.0% (16/50), and the DCR was 82.0% (41/50). In the CG, there were 3 (6.0%), 22 (44.0%), 20 (40.0%), 5 (10.0%), 50.0% (25/50), and 90.0% (45/50), respectively. ORR and DCR were markedly higher in the CG as against the TACEG (P <0.05) (Figure 3).

### Analysis of follow-up results of subjects

**Figure 4:**
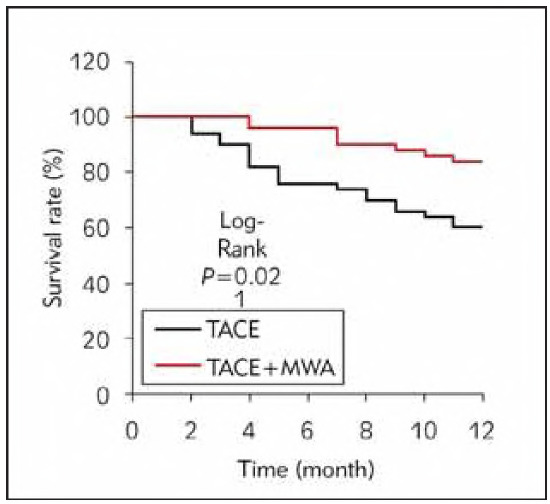
Contrast of Kaplan-Meier survival curves.

The OS of TACEG was 60.0% (30/50), and that of CG was 84.0% (42/50). The OS of the CG was markedly higher than that of the TACEG (P <0.05) (Figure 4).

### AR in subjects

**Figure 5:**
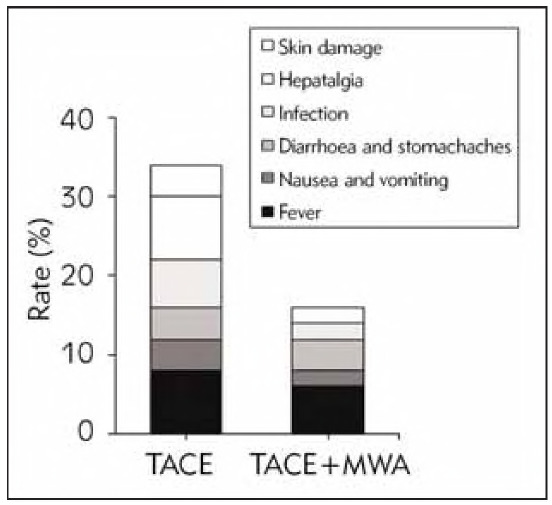
Contrast of AR.

In the TACEG, there were 4 cases (8.0%) of fever, 2 cases (4.0%) of nausea and vomiting, 2 cases (4.0%) of diarrhoea and stomachaches, 3 cases (6.0%) of infection, 4 cases (8.0%) of hepatalgia, and 2 cases (4.0%) of skin damage. The total AR rate was 34.0% (17/50). In the CG, there were 3 (6.0%), 1 (2.0%), 2 (4.0%), 1 (2.0%),0 (0.0%), 1 (2.0%), and the total AR rate was 16.0% (8/50). The incidence of AR was markedly lower in the CG as against the TACEG (P <0.05) (Figure 5).

### Analysis of 3 cases of skin injury

Case 1: Male, 47 years old, TNM stage IIIa PLC, Child-Pugh grade A, disease duration 11.8 months, AFP positive, 2 lesions, maximum diameter 3.36 cm. From June 2020 to October 2022, 6 TACE treatments were performed. Two hours after the seventh treatment, abdominal pain and a burning sensation occurred. After examination, it was found that there was a skin rash (3 x 3 cm on the abdominal skin), accompanied by apparent local redness, swelling, and pain. Symptomatic treatments such as local cold compress, external application of burn ointment, and antibacterial therapy were given in time. The symptoms of the subjects were markedly improved at discharge without infection, but there were still crusts and induration after palpation. After discharge, the patient continued to apply antibacterial and scar repair ointment and gradually recovered after 2 months.

Case 2: Male, 60 years old, TNM IIIa stage PLC, Child-Pugh grade B, disease duration 13.1 months, AFP positive, 3 lesions, maximum diameter 3.50 cm. The patient underwent hepatectomy in December 2020 and developed a recurrence of multiple nodules in the liver 3 months after surgery. From January 2021 to March 2022, the patient received 7 times of TACE + MWA treatments. Two hours after the 8th treatment, the patient developed abdominal pain and a burning sensation. After examination, it was found that there was a skin rash (4 x 4 cm on the abdominal skin) accompanied by obvious local redness, swelling, and pain. Symptomatic treatments such as local cold compress, external application of burn ointment, and antibacterial therapy were given in time. The symptoms of the subjects were markedly improved at discharge, without infection, with only dark brown pigmentation.

Case 3: Female, 52 years old, TNM stage 11 a PLC, Child-Pugh grade A, disease duration 5.4 months, AFP positive, 3 lesions, the maximum diameter of the lesions was 3.01 cm. From March 2021 to December 2021, a total of 3 times of TACE were performed. Two hours after the fourth treatment, the patient developed pain and a burning sensation in the upper abdomen. After examination, it was found that there was a skin rash (5 x 5 cm on the abdominal skin) accompanied by obvious pain. Symptomatic treatments such as local cold compress, external application of burn ointment, and antibacterial therapy were given in time. The symptoms of the subjects were markedly improved at discharge, without infection, with only dark red pigmentation.

## Discussion

PLC has a high degree of malignancy, and its onset is relatively insidious. Some patients are already in the middle and late stages when diagnosed. They lack the opportunity for surgical treatment, so the mortality rate is high [10] [11] [12]. Patients with middle or advanced PLC who do not have surgical indications need to receive chemotherapy or other treatments. TACE is considered the first choice for middle or advanced LC without surgical indications [13]. TACE achieves the purpose of therapy by injecting a mixture of embolic agents and chemotherapy drugs into the blood supply vessels of the target lesions through a catheter. As against the systemic chemotherapy remedy mode, TACE can increase the local concentration of chemotherapy drugs in the target lesions, reduce the drug concentration in the body circulation, and then reduce the toxic side effects caused by chemotherapy drugs [14]. However, the peripheral and fibrous capsule, extracapsular infiltrating tissue, and portal vein tumour thrombus of LC lesions are mainly supplied by veins, so the TACE remedy can't completely embolise the blood supply vessels of target lesions, and collateral circulation will be formed, resulting in reduced cancer cell killing efficiency [15] [16]. In addition, factors such as vascular occlusion, tumour ischemia, and hepatic arteriovenous fistula can also cause a rapid loss of chemotherapy drugs and affect the long-term survival of patients. When MWA is used in PLC, if the target lesion has less blood supply, the degree of heat energy loss during ablation remedy is lower, so the remedy has higher thermal efficiency and better therapeutic effect [17].

The decomposition products of cancer cells after degeneration and necrosis have certain antigenic characteristics, which can induce the generation of specific cytotoxic T cells and enhance the immune ability of cells [18]. As against normal cells, cancer cells are less tolerant to heat than normal cells, and local heating to 40-55 can inhibit the division of cancer cells and cause coagulative necrosis of target lesions [19]. This article found that after TACE alone and TACE plus MWA, the ORR of patients was 32% and 50%, the DCR was 82% and 90%, and the 1-year OS was 60% and 84%, respectively. TACE can reduce the target lesion's local blood supply and flow velocity and play a therapeutic effect [20]. MWA can expand the coagulation range of target lesions during remedy and markedly improve the tumour-killing effect. The heat generated during the remedy will strengthen the sensitivity of cancer cells to chemotherapy drugs [21]. Izzo et al. [22] suggested that the OS rate, local recurrence rate, complication rate, disease-free SR, and mortality rate of MWA for LC patients with focal lesions >3cm were 22 months, 5%, 17.8%, 2.2%, and 0%, respectively. MWA can reduce thermal injury during remedy and directly destroy the collateral circulation formed after TACE, thus further improving the therapeutic effect [23]. Therefore, the efficacy of TACE plus MWA is better.

This article detected liver function indexes in the peripheral blood of subjects. TBil is a product of the decomposition and destruction of senescent red blood cells in the monocyte-macrophage system, such as the liver. The liver plays a major role in bilirubin metabolism. When the liver has defects in bilirubin transport or binding, it will cause bilirubin metabolism disorders. Therefore, TBil and DBil are common liver function test indicators [24]. ALT is mainly found in the cells of the liver and heart tissue. In acute liver cell injury diseases such as acute viral hepatitis and drug-induced hepatocyte necrosis, ALP is released into the blood in large quantities [25]. AST is mainly found in the myocardium, skeletal muscle, and liver and increases when there is myocardial infarction, liver lesions, acute pancreatitis, and other diseases [26]. ALP is widely distributed in the liver and other tissues. It can catalyse the removal of the 5'-phosphate group from the nucleic acid molecule to convert the 5'-P terminal of the nucleic acid molecule into 5'-OH. GGT widely exists in the membrane and microsomes of liver cells, which participates in glutathione metabolism and is an important indicator for diagnosing liver function damage [27]. CG is conjugated bile acid formed by combining bile acid and glycine. The uptake ability of CG by hepatocytes decreases after injury, leading to increased CG levels in serum [28].

Hepatocytes mainly synthesise PAB and can be used in the auxiliary diagnosis of malnutrition, liver insufficiency, nephrotic syndrome, and other diseases [29]. AFP is a kind of serum glycoprotein, and the elevation of AFP is common in LC, gastric cancer, viral hepatitis, and other diseases. This article suggested that serum TBil, DBil, CG, and PAB were increased, while ALT, AST, ALP GGT, and AFP were decreased after TACE alone and TACE plus MWA. PAB<170 mg/dL is closely related to hepatic insufficiency after liver surgery [30]. This article suggested that the serum CG and PAB were markedly higher following remedy, while the serum TBil, DBil, ALT, AST, ALP GGT, and AFP were considerably lower in the CG as against the TACEG. AFP also promotes the immune escape of cancer cells by inhibiting tumour-infiltrating lymphocytes, natural killer cells, dendritic cells, and macrophages, so it is considered a target antigen for cancer immunotherapy [31]. Cancer cells killed by MWA can suppress the secretion of immuno-suppressive factors [32]. TACE can reduce the density of cancer tissue and the number of layers inside the lesion tissue, which is beneficial for uniform heat diffusion during MWA [33]. The results suggested improved liver function after TACE plus MWA was better.

This article suggested that the AR rate of TACE alone and TACE plus MWA was 34% and 16%, respectively. During TACE, tumour necrosis after ischemia and hypoxia will stimulate local nerves and cause pain symptoms. If the embolic agent leaks into the abdomen, it stimulates the peritoneum and nerves and causes abdominal pain [34]. In addition, TACE requires embolisation with embolic agents combined with chemotherapeutic drugs, and chemotherapeutic drugs can cause nausea and vomiting [35]. This article found that only 3 of the 100 patients had skin and subcutaneous tissue injury complications after surgery, and the patients suggested abdominal rash, local redness and swelling, pain, fever, etc. Complications of skin and subcutaneous tissue injury caused by TACE require close observation and active remedy [36]. Mild skin injury only requires local cold compress, topical burn ointment, etc., but there may be skin pigmentation, subcutaneous induration or scar, and other sequelae. Early local debridement is needed for severe skin injuries.

## Conclusion

The efficacy and safety of TACE plus MWA were better relative to TACE alone, the QoL of patients was markedly improved after the operation, and the short-term SR was higher. Skin and subcutaneous tissue injury complications after TACE and MWA were relatively rare, and symptomatic remedies can markedly improve the symptoms. This article only included 1-year follow-up data to evaluate the prognosis difference between TACE alone and TACE plus MWA. More follow-up time is needed for evaluation. This study provides a reference for selecting clinical remedy options and managing skin injury complications in middle or advanced PLC.

## Dodatak

### Acknowledgements

We would like to thank the staff and patients at Wuxi People's Hospital, Wuxi Medical Center, Nanjing Medical University, Wuxi, for their invaluable cooperation and support throughout the study.

### Conflict of interest statement

All the authors declare that they have no conflict of interest in this work.

### List of abbreviations

PLC, primary liver cancer;<br>TACE, transcatheter arterial chemoembolisation;<br>MWA, microwave ablation;<br>QoL, quality of life;<br>SF-36, short-form health survey with 36 items;<br>TBil, total bilirubin;<br>DBil, direct bilirubin;<br>ALT, alanine aminotransferase;<br>AST, aspartate aminotransferase;<br>ALP alkaline phosphatase;<br>GGT, glutamyl transpeptidase;<br>CG, cholyglycine;<br>PAB, prealbumin;<br>AFP alpha-feto-protein;<br>ORR, objective response rate;<br>DCR, disease control rate;<br>OS, overall survival;<br>CR, complete response;<br>PR, partial response;<br>SD, stable disease;<br>PD, progressive disease;<br>AR, adverse reactions;<br>SR, survival rate;<br>TNM, tumour, node, metastasis.
